# Minimum requirements for the design of Brazilian intensive care units: is it time for a change?

**DOI:** 10.62675/2965-2774.20260402

**Published:** 2026-05-22

**Authors:** Bruna Brandão Barreto, Mariana Luz, Patrícia Machado Veiga de Carvalho Mello, Dimitri Gusmao-Flores

**Affiliations:** 1 Hospital da Mulher Maria Luzia Costa dos Santos Intensive Care Unit Salvador BA Brazil Intensive Care Unit, Hospital da Mulher Maria Luzia Costa dos Santos - Salvador (BA), Brazil.; 2 Hospital de Terapia Intensiva Casamater Intensive Care Unit Teresina PI Brazil Intensive Care Unit, Hospital de Terapia Intensiva Casamater - Teresina (PI), Brazil.; 3 Centro Universitário UniFacid Wyden Teresina PI Brazil Centro Universitário UniFacid Wyden - Teresina (PI), Brazil.; 4 Universidade Federal da Bahia Faculdade de Medicina da Bahia Salvador BA Brazil Postgraduate Program in Medicine and Health, Faculdade de Medicina da Bahia, Universidade Federal da Bahia - Salvador (BA), Brazil.

**Keywords:** Intensive care units, Architectural drawing, Healing environment, Middle-income country

## Abstract

The Brazilian government's minimum requirements for intensive care unit design date back to 2002 and conflict with decades of scientific evidence that emphasize the environment's impact on patient healing and health care professionals’ performance and errors, jeopardizing patient, family member, and intensive care clinician outcomes. Using guidelines from the United States and Indian Society of Critical Care Medicine, the European Society of Intensive Care Medicine and the College of Intensive Care Medicine of Australia and New Zealand as comparators, this review showcase how Brazilian minimum requirements for intensive care unit design misalign with that scientific evidence, laying the groundwork for the development of evidence-based health policies by the national intensive care medicine society and government institutions. Five domains of intensive care unit design were addressed: patient visibility, bedside workspace, lighting, bedroom layout, and greenery and outdoor facilities. Under each domain, evidence is presented indicating that current national standards for intensive care unit design are associated with negative outcomes for patients, family members, and healthcare professionals, such as decreased safety for both patients and healthcare providers, delayed recovery, and increased work-related stress, absenteeism, and human errors. Therefore, updating Brazilian minimum requirements for intensive care unit design is an urgent and necessary step to improve critical care outcomes for patients, family members, and intensive care unit staff.

## INTRODUCTION

The intensive care unit (ICU) is a concept that emerged from the need to care for severely ill individuals who require constant vigilance and mechanical support with sophisticated devices, such as mechanical ventilators and hemodialysis machines. In this environment, specialized and dedicated personnel work 24/7, ensuring patient safety and promptly adjusting treatments based on the patient's condition. Intensive care clinicians must perform at their highest capacity, and their response to changes in a patient's condition must be immediate. Data used to evaluate patients’ status is gathered directly from the patient, through visual inspection and physical examination, and from various monitors and machines that continuously emit visual and auditory signals. This means intensive care professionals constantly process massive amounts of information for rapid decision-making, which depends on continuous interaction and interpretation between the healthcare worker and their environment.

**Figure 1:**
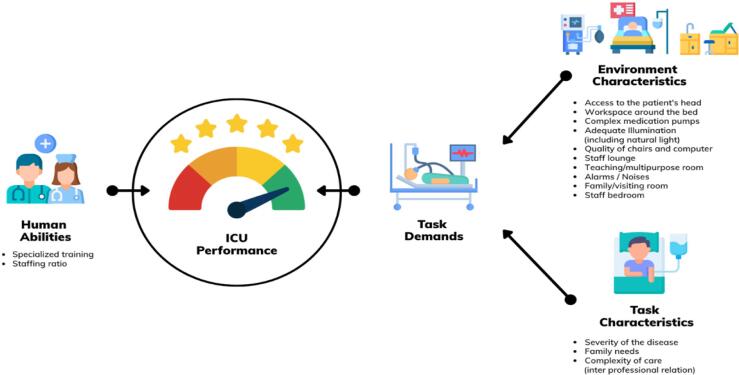
The performance of an intensive care team is influenced by the human abilities of the team members and the demands of the task they are required to perform.

The performance of an intensive care team is directly affected by the human abilities that compose this team and the demands of their tasks, which are influenced by the environment in which these tasks are performed (Figure 1).^(1,2)^ Although task characteristics are often a non-modifiable aspect of ICU care, many environmental characteristics can and must be optimized to increase the chances of a positive outcome for both patients and family members enduring critical illness. A positive outcome encompasses not only survival but also improved well-being and increased satisfaction with the ICU experience.^(3–5)^ The goal of this narrative review is to discuss these environmental characteristics in light of the Brazilian context by comparing current normative aspects of adult ICU architectural design with minimum standard recommendations worldwide, and providing a discussion for future aspects and concepts that should guide our path to the improvement of the intensive care workplace.

ICU - intensive care unit.

Source: based on the figure by Donchin et al.^(1)^

### Search strategy and selection criteria

Given the global relevance and leadership in critical care excellence, the recommendations from the United States Society of Critical Care Medicine (SCCM),^(6,7)^ the European Society of Intensive Care Medicine (ESICM)^(8)^ and the College of Intensive Care Medicine of Australia and New Zealand^(9)^ were established as the primary comparators to the current Brazilian governmental recommendation, specifically the *Agência Nacional de Vigilância Sanitária* (ANVISA) Collegiate Board Resolution (*Resolução da Diretoria Colegiada* - RDC) n° 50, published on February 21, 2002.^(10)^ Additionally, from a socioeconomic perspective, the Indian Society of Critical Care Medicine (ISCCM) guideline^(11)^ was chosen as a comparator from a middle-income country.

A literature search was conducted using the MEDLINE® (Ovid) database, combining the terms "adult intensive care unit", "design", "architecture", and "environment". No language or date restrictions were applied to ensure a comprehensive capture of relevant studies.

Furthermore, the reference lists of the society guidelines, retrieved articles, and relevant review papers were manually screened to identify additional potentially eligible studies. The selection of articles for inclusion was based on their relevance to the scope of this review, focusing on studies that elucidated the impact of the ICU environment on the outcomes of patients, family members, and health care professionals.

After a round of discussion among the authors, five topics in ICU design were identified as relevant for an initial investigation: patient visibility, bedside workspace, lighting, bedroom layout, and greenery and outdoor facilities. The first four domains were chosen for their direct impact on bedside critical care delivery. At the same time, greenery and outdoor facilities were selected for their relevance to contemporary discussions on healing environments. Additional areas of ICU design-relevant research are noted and may drive future inquiry. An example of a Brazilian public ICU floor plan is provided in the figure 1S (Supplementary Material).

### Creating a healing environment in the intensive care unit

Since the interaction among humans, machines, and the environment is central to ICU care, and given that ICU workers spend 8 to 12 hours in the unit, ICU design should be primarily driven by its function.^(12)^ Using the framework proposed by Avedis Donabedian,^(13)^ to provide high-quality critical care, the ICU configuration (structure) should be planned to facilitate human work (work process), taking into consideration its specifics needs and capacities, This approach aims to increase performance, minimize human errors,^(14)^ and ensure safety, all while preserving the well-being of both patients and professionals (expected outcomes).

Human-centered design (HCD) is an approach that prioritizes human needs, capabilities, and behaviors, designing to accommodate them.^(15)^ In the context of the ICU, both patients’ and workers’ needs must be considered to optimize patient care. Since healthcare workers constantly interact with the environment to provide care, it is logical that an environment that makes this task more difficult can significantly impact patient outcomes. Therefore, an adequate ICU design must consider the perspective and needs of intensive care clinicians. Excellent care will hardly be achieved with dissatisfied staff. Despite the growing knowledge on the importance of healthcare professionals’ well-being as a way to improve patient-centered outcomes,^(16)^ in practice, "the gap between our concern for treating the patient and the attention paid to the worker's needs is too big", as stated by Donchin et al.^(1)^

Currently in Brazil, the standard design for opening an ICU is set by the RDC 50/2002.^(10)^ However, this resolution fails to consider the needs of intensive care in critical aspects, such as workspace area, access to the head of the bed, illumination, and patient visibility by the healthcare team. Furthermore, the resolution does not adequately address the high-pressure context of ICU work. It therefore does not sufficiently account for the need for areas that can alleviate the stress and emotional tension experienced by ICU staff, which will be addressed in the section on greenery and outdoor facilities.

### Visibility

The "2024 Guidelines on ICU Design" issued by the SCCM strongly recommended high-visibility ICUs. Intensive care clinicians should be able to see the patient's face, monitors, and bedside alarms from the staff workstation, as constant monitoring of patients at high risk of deterioration is a fundamental aspect of ICU care.^(6)^ This concept is also clearly stated in the 2010 Guidelines for Design and Construction of Health Care Facilities from The Facility Guidelines Institute (FGI)*,* which states that observation from nursing stations "shall provide a view of the patient while the patient is in bed". In its "Recommendations on basic requirements for intensive care units: structural and organizational aspects", the ESICM recommends that "the patient should be able to be visualized at all times to facilitate detection of status changes and enhance implementation of therapeutic actions" and this visualization should be achieved by "large window openings, glass doors etc".^(8)^ This is also the recommendation of the College of Intensive Care Medicine of Australia and New Zealand^(9)^ and the ISCCM guideline.^(11)^ These documents also report the common idea that there should be ways to ensure patients’ privacy when needed, but without jeopardizing patient safety.

**Figure 2:**
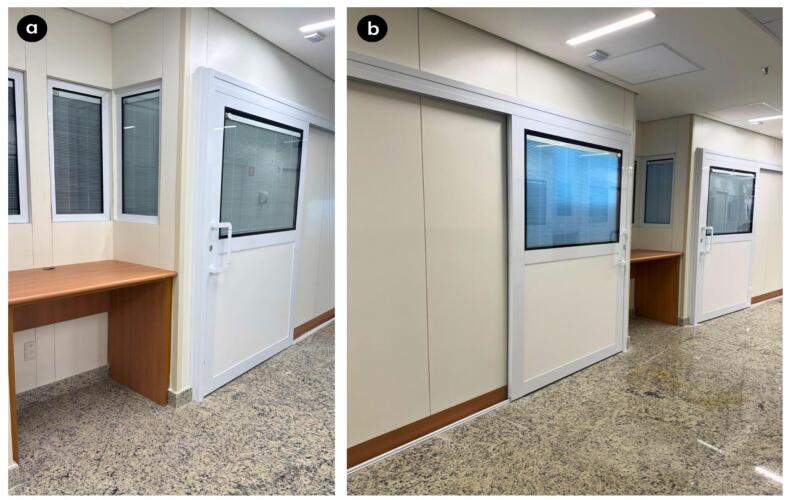
Example of a low-visibility intensive care unit bedroom in a private hospital.

The RDC 50/2002,^(10)^ on the other hand, allows direct patient visualization to be replaced by monitor visualization at a central monitoring station. This flexibility, combined with the increasing adoption of hotel-like hospitality concepts that prioritize privacy, especially in Brazilian private hospital chains, enables the construction of ICU beds with minimal or no visual contact with patients. This can be due to opaque doors with low-visibility windows (Figure 2) or beds located far from the nursing workstation, where constant monitoring relies solely on central monitor stations displaying vital signs or requires long walks to access the bedside.

(A) A patient can be seen only through the small window at the door, and the view through this window is obstructed by blinds. (B) A partial view of the corridor with low-visibility single-bed cubicles.

The ability of ICU clinicians to visually monitor patients is associated with increased perception of safety,^(17)^ improved staff communication,^(18,19)^ reduced staff stress,^(20)^ and positive patient outcomes such as decreased falls and mortality.^(21,22)^ There is a growing body of literature discussing how different ICU layouts and typologies can impact patient visibility.^(23,24)^ Critically ill patients and their bedside monitors should be within the visual field of intensive care nurses and physicians from the working station at all times. Central monitors do not replace constant visual inspection and physical examination. High visibility should be prioritized in future Brazilian guidelines for ICU design.

### Bedside workspace

Critical aspects of bedside design that directly impact patient care include: safe and easy access to the patient, the possibility of easily and quickly accessing the patient's head in case of urgent need to control the airway, and sufficient space to accommodate machines, monitors, and allow for sterile bedside procedures without contamination risk. The RDC 50/2002 recommends a patient space of 10.0m^2^, whereas the FGI recommends at least 18.6m^2^ (with a minimum of 14m^2^ for existing units where renovation is not yet permitted). The College of Intensive Care of Australia and New Zealand^(9)^ and the ESICM^(8)^ recommend a minimum of 20.0m^2^ for common rooms and 25.0m^2^ for single rooms, with at least 2.5m of traffic area beyond the bed area. The ISCCM recommends at least 14.0m^2^ to 18.0m^2^ for common areas and 18.0m^2^ to 23.0m^2^ for single-bed cubicles.^(11)^

**Figure 3:**
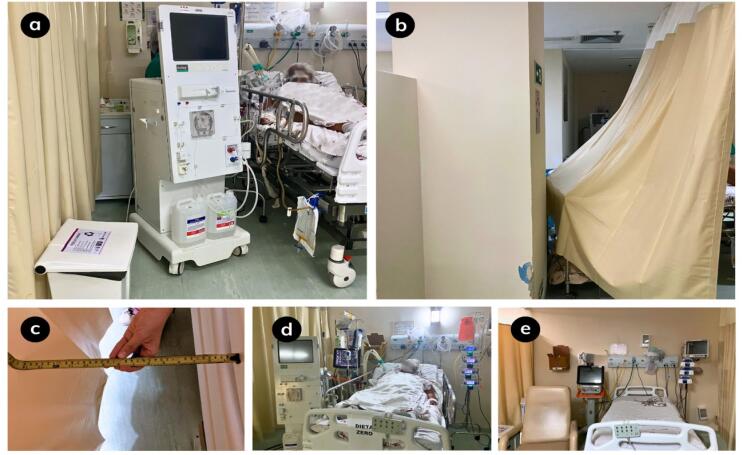
The risks and obstacles associated with an undersized workspace.

**Figure 4:**
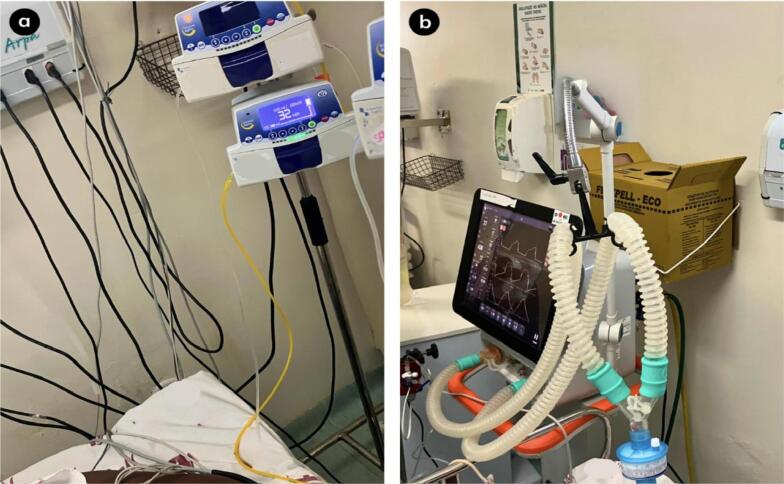
The access to the head of the bed should be easy, allowing prompt performance of life-saving interventions.

An undersized room precludes routine care such as mobilization, increases the workload due to the constant movement of heavy equipment to provide care, and is considered a safety risk.^(25,26)^ Therefore, the Brazilian standard recommendation creates a difficult environment for performing procedures and attending to patients while protecting patient privacy, as the "closed" room area frequently does not accommodate the machines, tables, and other materials required for procedures, leading to situations such as those depicted in figure 3A. The workspace area shown in the figure is even smaller than the RDC recommends, at 8.9m^2^. Consequently, access to the head of the bed is also compromised, as the bed must be constantly against the wall, and circuits and wires from mechanical ventilators and bedside monitors obstruct the lateral passages needed for orotracheal intubation and sterile procedures, such as urgent intravenous line placement (Figure 4A).

(A) The hemodialysis machine is positioned in front of the mechanical ventilator, obstructing access to it in case of emergency and preventing visualization of the respiratory support graphs. (B) The area around the bed is inadequate for performing high-risk procedures with privacy, such as the placement of a central venous access, as the equipment cannot be secured within the limits of the workspace without risking contamination or limiting access to the patient. (C) The minimal space between the foot of the bed and the curtain (37cm) impedes effective cardiopulmonary resuscitation maneuvers. (D) Due to the small workspace area, access to the head of the bed is obstructed, which delays care in case of urgent airway management (accidental extubation, for example). It is not possible to keep family members at the bedside without further compromising access to the critical care bed. (E) The recliner chair for family members is also part of the workspace area, but its position must be carefully planned in light of the reduced workspace area.

Instead, in Brazil, the access to the head of the bed is frequently obstructed by medication lines and electrical cables (A), sharp containers and circuits from the mechanical ventilator (B).

The presence of multiple electrical connections tethered to fixed wall outlets also creates barriers to accessing the head of the bed, potentially delaying life-saving interventions (Figure 4B). To address this limitation, contemporary ICU designs increasingly incorporate mobile and/or suspended poles equipped with electrical outlets. By relocating essential connections – such as those for mechanical ventilators and infusion pumps – onto these poles, at least one side of the head of the bed can remain unobstructed. This configuration facilitates immediate access for physicians performing emergency procedures and should be considered the current gold standard in ICU infrastructure.

Considering that a "family zone" is also recommended^(27)^ and that loved ones are an important part of patient care, the lack of space is an obstacle to the constant presence of family members.^(26,28)^ Brazilian future guidelines should recommend a minimum of 18.0m^2^ of bedside workspace.

### Lighting

The "block" design of ICUs, characterized by internal, windowless units, remains common in Brazil (Figure 2SB in the Supplementary Material). The negative aspects of this type of design for both patients and healthcare professionals have been reported in literature for over 50 years.^(29)^ Beyond more exposure to daylight in terms of illuminance, the perceived change in light colors during the diurnal cycle is also crucial for human health (i.e., white light during daytime, warm-yellow light late in the evenings and night), as a fixed illumination pattern alters the natural circadian rhythm and melatonin secretion.^(30)^

Patients exposed to natural light also experience less perceived stress, use fewer analgesics, and have improved sleep quality.^(31–33)^ In patients receiving mechanical ventilation, the exposure to natural light through windows was associated with fewer episodes of hallucination and agitation requiring intervention with antipsychotics.^(34)^ In patients with prolonged mechanical ventilation, direct sunlight exposure was associated with faster weaning time.^(35)^ In the subgroup of illiterate patients, there was a significant decrease in length of stay and hospital costs, which should be further evaluated, especially considering the population admitted to the Brazilian public health system.^(36)^ One study in a cardiac ICU also suggested a small but significant reduction in the length of stay.^(37)^ It also suggested that the level of access to daylight and views, due to the bed orientation toward the window. may interfere with those outcomes.

The impact of natural daylight on delirium outcomes is unclear. While some studies show that the absence of windows and/or visible daylight is associated with a higher risk of delirium episodes,^(38–42)^ others have not found this association.^(43–46)^ Moreover, in some studies, the impact of window and daylight exposure on delirium outcomes cannot be separated from a multicomponent intervention, such as single-bed rooms and noise reduction.^(47)^

For staff, the presence of windows and daylight is associated with improved job satisfaction, decreased sleepiness and stress, improved mood, increased communication, and decreased absenteeism.^(31,48,49)^ In a before-and-after study, the use of dynamic lighting was associated with improved mood, reduced fatigue, and better sleep quality compared to "on/off" fluorescent halogen lamps.^(50)^ Additionally, exposure to high illuminance lighting during night shifts is associated with increased psychomotor errors.^(51)^

People prefer daylight over electric lighting, and lighting is frequently attributed as a source of symptoms of sick-building syndrome.^(52)^ While studies on this topic are observational in nature, which downgrades the certainty of the evidence regarding clinical outcomes, the presence of windows and natural lighting is considered to play an important role in the humanization of a space, with a meaningful impact on satisfaction of both patients and healthcare professionals. For this reason, it is consistently recommended in the minimum standards of the European, Indian, and Australian/New Zealand ICUs, and is strongly recommended by the SCCM guideline (Table 1).^(6–9,11,53)^ In contrast, the RDC 50/2002 does not clearly recommend natural daylight in the ICU, nor does it include recommendations regarding windows. Therefore, it is very common in Brazil that the workspace area in the ICU (i.e., patient's bedroom and central nursing station) is illuminated solely by overhead artificial "on/off" fluorescent or light-emitting diode (LED) lamps (Figure 2SC - Supplementary Material) with cool white temperature (6,500 Kelvin), without natural light exposure or windows. The presence of window views and natural lighting in the ICU workspace area should be clearly established as a standard in future Brazilian guidelines.

**Table 1:** Comparative analysis: RDC 50/2002 *versus* international guidelines

Aspect	Brazil*	United States†	Europe†	Oceania§	India¶
Patient visibility	Not addressed	High-visibility	Patients should be visualized at all times (large window opening, glass doors)	High-visibility; direct patient observation from nursing stations	Patient must be easily visible from the charting area
Window and natural light	Not addressed	Yes	Yes	Yes	Yes
Room size	10.0 m² with a distance of 1 m from the lateral walls, except for the head and foot of the bed, which must be at least 1.2 m away from the wall	FGI 2010: minimum 18.6m^2^ with a minimum headwall width 3,96m per bed SCCM 2012: clear area around the standard critical care bed, distancing not less than 1,2m from the head and foot of the bed, and not less than 1,8m on each side (this does not include space needed for staff and family support functions)	Minimum of 25m^2^ for single rooms and 20m^2^ per bed for common rooms, rectangular with at least 2.5m traffic area beyond the bed area	Minimum of 25 m^2^ for single rooms and 20 m^2^ per bed for common rooms	At least 14m^2^ or 18m^2^ for open-bay layout. 18 to 23m^2^ for single bedrooms. The isolation room should have 20% extra space
Nursing station	At least one of the stations (when there is more than one) must have 6.0m²	Centralized or decentralized	Central	Central	Central
Medical prescription room/medical office	1.5m^2^	1m^2^ to serve every two beds	20m^2^ (medical office)	Yes	Yes
Family interview room/ consultation room	6.0m^2^	Yes (capacity of not less than 1,5 persons per patient bed)	15 m^2^ room	Yes, with facilities to deal with more than one grieving family	Yes
Kitchen	2.6m² with a minimum dimension of 1.15m	Yes	(in the staff lounge)	Yes	Not addressed
On-call bedrooms	2.6m² with a minimum dimension of 1.15m	Yes	15 m^2^ room with a bed, a hand-basin, a shower, a toilet, a telephone, a television, and access to daylight with an operable window	Yes	Not addressed
Staff lounge	1.3m^2^ per person	Yes (separate from multipurpose/teaching room) - minimum of 9.29m^2^	A staff lounge of 40 m^2^ for eight intensive care beds	Yes	Yes
Family accommodation	Not addressed	Yes	Yes	Yes	Yes
Teaching / multipurpose room	Not addressed	Yes	40 m^2^ space for formal teaching	Yes	Yes

To further highlight the discrepancies between Brazilian regulations and international recommendations, the table provides a detailed comparison of key aspects of intensive care unit design.

* Resolução da Diretoria Colegiada n° 50/2002;

† SCCM 2024 Guidelines on ICU Design/SCCM Guidelines for intensive care unit design 2012 / FGI 2010; † European Society of Intensive Care Medicine recommendations on basic requirements for intensive care units: structural and organizational aspects 2011;

§ College of Intensive Care Medicine of Australia and New Zealand 2016;

¶ Indian Society of Critical Care Medicine Experts Committee Consensus Statement on ICU Planning and Designing, 2020. FGI - Facility Guidelines Institute; SCCM - United States Society of Critical Care Medicine.

Most ICUs in contemporary centers of excellence outside of Brazil are designed not only to incorporate large windows at the bedside, but also to position them laterally (Figure 2SA - Supplementary Material). This arrangement ensures that patients are exposed to natural daylight while also being able to view the external environment – whether it be buildings, the sky, or surrounding landscapes. In contrast, the majority of Brazilian ICUs, even when equipped with windows, are typically small and placed behind the head of the bed. As a result, while natural light may enter the room, patients are deprived of any meaningful visual connection with the outside world.

Modern ICU design trends often position administrative and staff work areas at the core of the unit's floor plan, situating patient rooms along the perimeter. This configuration guarantees that each room has direct access to a window, allowing patients to visually engage with the external environment while maintaining visibility into the patient rooms.

### Greenery and outdoor facilities

Immersion in nature has a profound emotional impact, not only increasing positive affect (such as joy and excitement) but also decreasing negative affect (such as fear, guilt, anger, or sadness),^(54)^ with a well-known impact on different health outcomes.^(55,56)^ For this reason, nature exposure is an important aspect to consider when designing a healing environment,^(57)^ with increasing attention given to the benefits of greenery and outdoor spaces for critically ill patients, their families, and the healthcare professionals working in the ICU.^(58–60)^

For patients, viewing nature was associated with a shorter hospital stay, reduced analgesic use, and nurses’ notes reporting more positive mood and behavior during recovery.^(32)^ Natural landscape may decrease pain by eliciting positive emotions, reducing stress, and distracting patients from focusing on their pain.^(31)^ It is also associated with decreased stress, improved coping, and increased spiritual connection, thereby enhancing patients’ and families’ satisfaction, whether adults or children.^(61)^ For family members of critically ill patients, breaks spent in the hospital garden decreased sadness compared with other indoor locations.^(62)^

Exposure to nature is also beneficial for staff, as it is associated with reduced stress and increased satisfaction.^(31)^ Nurses who rested for at least 15 minutes in a hospital-integrated garden experienced less emotional exhaustion and depersonalization after a period of 6 weeks, and immediate improvements in feelings of anger and tiredness.^(63)^

Although the provision of a view of a natural landscape is suggested by the SCCM guideline^(7)^ and mentioned, but not emphasized as required, in the ISCCM guideline,^(11)^ it is not addressed in the ESICM guideline^(8)>^ or the RDC 50/2002.^(10)^ Easy access to gardens and outdoor facilities should be emphasized in future Brazilian guidelines. There is a growing body of literature addressing the design of healing gardens.^(64,65)^

### Single-bed room versus open bay layout

Although the common rooms are an option in the documents from the ESICM, ISCCM^(11)^ and the College of Intensive Care Medicine of Australia and New Zealand,^(9)^ the ESICM issued a strong recommendation for single-bed rooms^(8)^ due to the likely reduction in incidence of infections and delirium,^(66)^ as well as increased patient and family satisfaction.^(67)^ The SCCM guideline,^(6)^ however, issued a conditional recommendation for single-bed rooms, which was directly related to decreased visibility of patients compared to an open layout.

The RDC 50/2002^(10)^ leaves the decision regarding a single-bed or open-plan unit to the discretion of the healthcare facility, and, with the increase in hotel-like hospitality, particularly in private hospitals, the adoption of single rooms in Brazilian ICUs has increased. However, there are two disadvantages of the single-bed design that are particularly worrisome when considering ICU care in Brazil.

First, single-bed rooms are associated with a perceived decrease in patient safety,^(25,68,69)^ with an increased use of physical restraints,^(68)^ increased stress for healthcare professionals,^(67,68,70)^ increased walking distance to the patients’ bedside,^(68)^ which is associated with less time with patients,^(71)^ and decreased teamwork and communication.^(68,69)^ While this perception of increased risk was documented in ICUs with a nurse-to-patient ratio of 1:1 to 1:2,^(25,68,69)^ it is important to note that the minimum standard for Brazilian ICUs is a nurse-to-patient ratio of 1:10 and a physician-to-patient ratio also of 1:10. This, coupled with the low-visibility characteristics of many current single-bed rooms, greatly contributes to the decrease in patient safety. Therefore, the interplay between single-bed rooms, visibility, and staffing ratio should be considered when planning an effective ICU.

Second, although there is evidence of increased patient and family satisfaction with single-bed rooms, many patients also prefer open-bay areas to avoid isolation and loneliness.^(72)^ Working in the Brazilian public healthcare system, this request is not uncommon, considering that many families are unable to provide company to their sick loved ones who frequently receive treatment outside their city of origin due to socioeconomic factors.

Therefore, we believe that a mixed approach to ICU layout, with at least three of ten beds as single-bed rooms and the remaining beds maintained in an open-bay layout, would allow for more individualized patient care. In ICUs with only one nurse and physician for every ten beds, critically ill patients requiring organ support (mechanical ventilation, hemodialysis) and constant vigilance/monitoring should be cared for in the open-bay area. This layout facilitates better communication among staff and when calling for help, is associated with greater safety for both patients and staff, and allows more than one patient to be observed at a glance, regardless of the staff's position within the unit. Single-bed rooms should be reserved for patients in recovery or those with a lower risk of deterioration (for instance, elective surgical patients or those requiring a lower level of care, such as low-flow oxygen supplementation). Those patients could benefit from increased privacy, especially when accompanied by family members who can contribute to vigilance for communicative patients, provided that anticipated causes of deterioration are easily recognized by individuals unfamiliar with healthcare.

The adoption of any structural standards must be accompanied by a thorough analysis of work processes and expected outcomes. For instance, the implementation of single-bed rooms (structure), which is internationally advocated to reduce infections and delirium (outcomes), may compromise or even reverse its benefits if not accompanied by adequate processes, such as sufficient nurse-to-patient ratios and high-visibility solutions. In the Brazilian context, where the nurse-to-patient ratio is often 1:10 (process), adopting closed rooms without process modifications may isolate patients and hinder monitoring. Therefore, future Brazilian guidelines must be systemic, specifying not only square footage or door type, but also how the structure must interact with minimum care processes to achieve the desired outcomes in safety, comfort, and efficiency.

### Practical and economic implications

It is well known that the Brazilian national healthcare system is underfunded by the Brazilian government. Brazil is one of the countries with the lowest relative public expenditure in healthcare among Organization for Economic Co-operation and Development (OECD) countries, investing only 4.0% of its gross domestic product (GDP), compared with 5.5% coming from the private sector.^(73)^ Regional inequities also exist, with the state of Bahia being one of the states with the lowest healthcare investments.^(74)^ Therefore, increasing healthcare investments originating from public funds is long overdue.

The proposed improvements in ICU design, while seemingly focused on physical infrastructure, carry significant practical and economic implications that extend beyond immediate construction costs. Enhancing patient visibility, providing adequate bedside workspace, ensuring natural light exposure, and integrating greenery can lead to tangible benefits:

– Improved patient safety and outcomes: a well-designed ICU environment, with features like high visibility and sufficient workspace, directly contributes to reducing medical errors and improving the efficiency of critical interventions. This can lead to shorter lengths of stay and reduced complications, ultimately lowering overall healthcare costs.
– Enhanced staff well-being and performance: the high-stress nature of ICU work often leads to burnout and decreased job satisfaction among healthcare professionals. Environments that incorporate natural light, views of nature, and dedicated respite areas can significantly mitigate these issues. A more rested, less stressed, and more satisfied staff is more productive, makes fewer errors, and is less likely to experience absenteeism, leading to improved quality of care and reduced staff turnover costs.
– Reduced hospital costs: as noted in the discussion on lighting, studies have shown that exposure to natural light can decrease hospital stays and reduce costs, particularly for certain patient populations.^(36)^ While the initial investment in design improvements might seem substantial, the long-term savings from improved patient outcomes, reduced readmissions, and a more stable workforce can be considerable.
– Increased patient and family satisfaction: a healing environment that considers the needs of patients and their families, including privacy, comfort, and access to nature, can significantly enhance their overall experience. This not only contributes to better psychological outcomes but also positively impacts the hospital's reputation and patient loyalty.

These implications underscore the importance of viewing ICU design not merely as a structural requirement but as a strategic investment in patient care quality, staff retention, and financial sustainability.

### Unaddressed aspects of intensive care unit design

Innumerable aspects of ICU design were not explored in this manuscript such as ICU typologies (radial, straight, pods, other), family accommodation and consultation room, staff respite area, decentralized versus centralized station, sound management, ICU logistics and supplies, ICU pharmacy, location of administrative leadership space and education supporting conference room, use ergonomic equipment, among others. Due to the scope of the task, it is urgent that the *Associação de Medicina Intensiva Brasileira* (AMIB) lead the way in rethinking ICU design and updating the minimum requirements for Brazilian ICUs in line with the extensive body of evidence from decades of research.

### Limitation

First, this study is a narrative review, and due to the nonsystematic nature of this research, the evidence presented here is susceptible to author selection bias. Second, although the five selected themes were defined prior to literature research and after thorough discussion among the authors, who have more than a decade of experience in intensive care, they may not reflect design priorities in every ICU. Third, most of the evidence presented here comes primarily from high-income countries, and changes here proposed should be planned with consideration for the unique funding and managerial challenges of the Brazilian public health system.

## CONCLUSION

This narrative review highlights critical discrepancies between current Brazilian regulations (RDC 50/2002) for intensive care unit design and international best practices. Key areas of concern include patient visibility, bedside workspace dimensions, natural lighting, and the provision of greenery and outdoor facilities. While the RDC 50/2002 offers a foundational framework, it falls short in addressing modern intensive care needs, particularly in its failure to prioritize human-centered design principles that prioritize the well-being of both patients and healthcare professionals. The lack of clear guidelines for high-visibility intensive care units, adequate bedside space, natural light exposure, and staff respite areas can negatively impact patient safety, staff performance, and overall intensive care units’ experience. Updating Brazilian guidelines to align with international standards is crucial for improving the quality of care, reducing medical errors, enhancing staff satisfaction, and ultimately, achieving better outcomes for critically ill patients in Brazil. This requires a comprehensive revision that incorporates evidence-based design principles and acknowledges the complex interplay between the physical environment and the human elements of intensive care.

## Data Availability

The data cannot be made publicly available because it is a narrative review.
