# Supplementary material for: Minimum requirements for the design of Brazilian intensive care units: is it time for a change?
Source: Crit Care Sci. 2026 May 22;38:e20260402. doi: 10.62675/2965-2774.20260402 (PMC13399251; doi:10.62675/2965-2774.20260402)
Supplement: Supplementary Material [file 2965-2774-ccsci-38-e20260402-suppl01.pdf]

Critical Care Science
https://doi.org/10.62675/2965-2774.20260402

SUPPLEMENTARY MATERIAL

Minimum requirements for the design of Brazilian intensive care units: is it time for a change?

Bruna Brandão Barreto[1], Mariana Luz[1], Patrícia Machado Veiga de Carvalho Mello[2,3], Dimitri Gusmao-Flores[1,4]

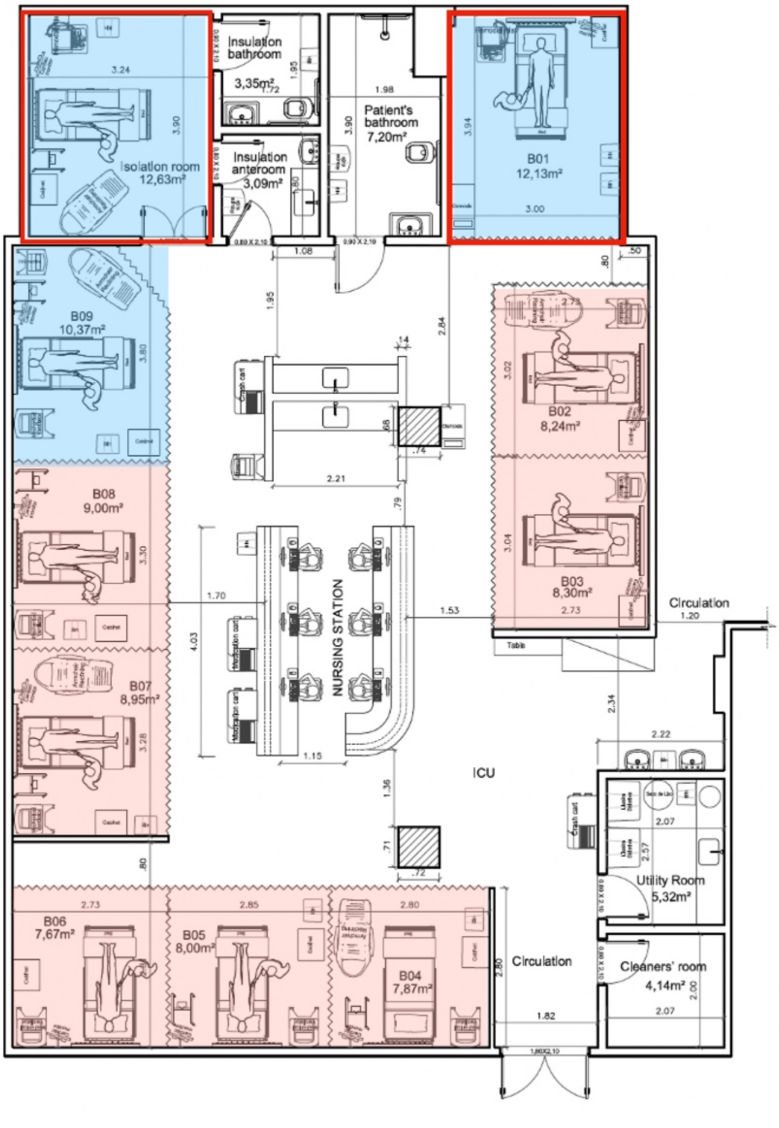

ICU - intensive care unit.

Figure 1S - Example of a floor plan of a Brazilian public intensive care unit (patient care area only).

Positive aspects: This is a representation of an open-bay intensive care unit with high visibility of most beds, with only B01 and B10, the isolation room, having low-visibility (red squares). Nursing station is centrally located, allowing a broad view of the unit, which facilitates monitoring of highly unstable patients and helps identify healthcare professionals who are in need of help during patient care.

Negative aspects: the seven rooms have undersized workspace areas for *Resolução da Diretoria Colegiada* (RDC) standards (shaded red), with the remaining three being undersized under international standards (shaded blue), with areas ranging from 7.7m2 to 12.6m2. This adds difficulty to the care of a critically ill patient in need of multiple organ support, decreasing the safety of both patients and health care professionals. The presence of a recliner for family members further decreases the patient and caregiver zones. The reduced workspace area together with the open bay intensive care unit plan result in a decrease of patients' and family members' privacy. There are no windows, which means that there is no access to daylight or external view.

This is an open access article under the CC BY license (https://creativecommons.org/licenses/by/4.0/).

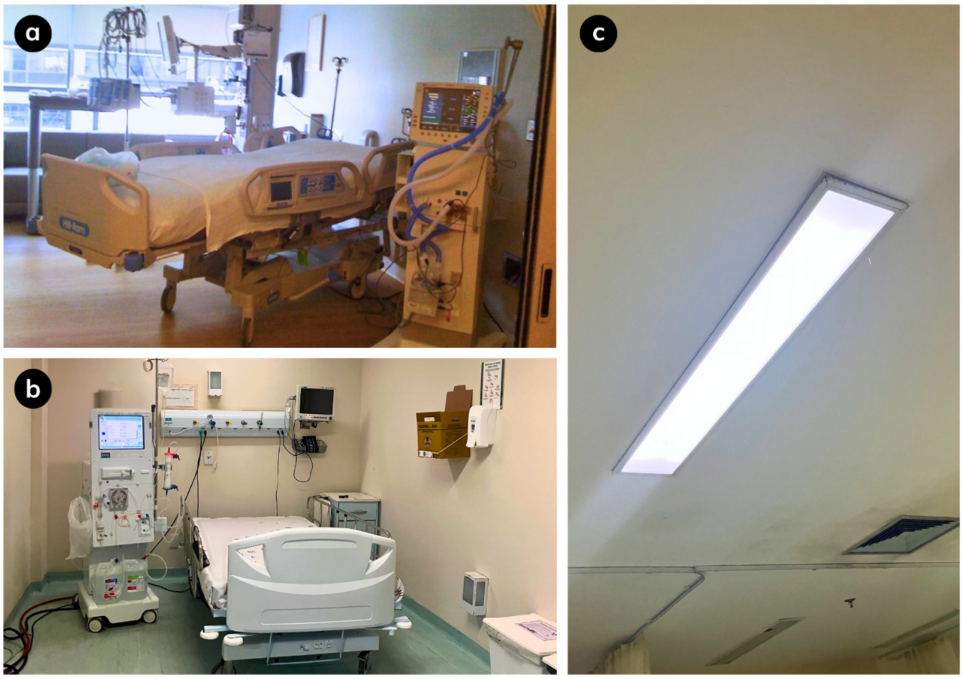

Figure 2S - Intensive care unit lighting.

A) Example of natural daylight illumination through a large lateral window in an intensive care unit outside of Brazil. B) Windowless intensive care units are still very common in Brazil. C) Overhead on/off light-emitting diode luminaires (cool white – 6500Kelvin) are frequently the only source of light in Brazilian intensive care units.
